# Effect of advancing paternal age on semen parameters and seminal oxidative stress markers in infertile men

**DOI:** 10.1186/1471-2164-15-S2-P42

**Published:** 2014-04-02

**Authors:** Saad Alshahrani, Ashok Agarwal, Mourad Assidi, Adel M Abuzenadah, Damayanthi Durairajanayagam, Ahmet Ayaz, Rakesh Sharma, Edmund Sabanegh

**Affiliations:** 1 Center for Reproductive Medicine, Cleveland Clinic, Cleveland, Ohio 44195, USA; 2 Salman Bin Abdulaziz University, College of Medicine, Saudi Arabia; 3 Center of Excellence in Genomic Medicine Research, King Abdulaziz University, Jeddah, Saudi Arabia; 4 KACST Technology Innovation Center for Personalized Medicine at King Abdulaziz University, Jeddah, Saudi Arabia; 5 MARA University of Technology, Sungai, Selangor Darul Ehsan, Malaysia

## Background

High rate of subfertility and adverse pregnancy related outcomes associated with childbearing are seen after age 40. In contrast to oogenesis, spermatogenesis continues in elderly men. Adult male germ cells pass through significantly more mitotic replications than the germ cells in adult female. In men, there is an age associated increase in the incidence of breaks in sperm DNA, decrease in apoptosis, and a higher frequency of point mutations. Advanced paternal age is associated with an increased time to pregnancy and decreased pregnancy rates. After adjusting for female age, conception during a 12-month period was > 30 percent less likely for men over 40 years of age as compared to men < 30 years of age [1]. Similarly, a five-fold increase in time to pregnancy was reported in men >45 years compared to men <25 years of age [2]. Age of the husband was the most significant factor contributing to a decreased probability of a pregnancy [3]. Advanced paternal age may result in congenital anomalies in progeny due to an increase in new autosomal dominant mutations such as achondroplasia; Apert, Waardenburg, Crouzon, Pfeiffer, and Marfan syndromes [4]. The goal of our study was to investigate the impact of male ageing on semen quality and seminal oxidative stress (OS) markers.

## Materials and methods

In this study, we examined the medical records of 472 infertile men referred to our laboratory between 2008 and 2012. Based on their age, patients were divided into group 1: ≤ 30 years (n = 69); group 2: 31-40 years (n = 298); and group 3:> 40 years (n = 105). We evaluated the conventional semen parameters (WHO, 2010) [5] and OS markers: seminal ROS (chemiluminescence assay), total antioxidant capacity (TAC) and sperm DNA damage (TUNEL assay) in all patients.

## Results

The mean age of the study subjects was 36.8 ± 6.7 years. No age-related differences were seen in conventional semen parameters (volume, concentration, motility, and morphology) (Table 1). ROS and antioxidant levels were comparable in the 3 groups. Significantly higher levels of sperm DNA damage (19.94 ± 15.30%) was seen in infertile men >40 years compared to men in younger age groups (P = 0.028 and P = 0.027, respectively).

## Conclusions

Sperm DNA damage increases with advancing paternal age. Evaluation of sperm DNA damage will help diagnose the underlying cause of poor fertility in some men and assist the clinician in offering correct treatment modality.
